# Cross-species genomics identifies DLG2 as a tumor suppressor in osteosarcoma

**DOI:** 10.1038/s41388-018-0444-4

**Published:** 2018-08-09

**Authors:** Yang W. Shao, Geoffrey A. Wood, Jinchang Lu, Qing-Lian Tang, Jonathan Liu, Sam Molyneux, Yan Chen, Hui Fang, Hibret Adissu, Trevor McKee, Paul Waterhouse, Rama Khokha

**Affiliations:** 1 Princess Margaret Cancer Centre, University of Toronto, Toronto, Ontario M5G 1L7 Canada; 2 Department of Pathobiology, Ontario Veterinary College, University of Guelph, Guelph, Ontario N1G 2W1 Canada; 3 Department of Orthopedic Oncology, the First Affiliated Hospital, Sun Yat-Sen Univeristy, 510080 Guangzhou, China

**Keywords:** Bone cancer, Cancer genomics

## Abstract

Leveraging the conserved cancer genomes across mammals has the potential to transform driver gene discovery in orphan cancers. Here, we combine cross-species genomics with validation across human–dog–mouse systems to uncover a new bone tumor suppressor gene. Comparative genomics of spontaneous human and dog osteosarcomas (OS) expose Disks Large Homolog 2 (DLG2) as a tumor suppressor candidate. DLG2 copy number loss occurs in 42% of human and 56% of canine OS. Functional validation through pertinent human and canine OS *DLG2*-deficient cell lines identifies a regulatory role of DLG2 in cell division, migration and tumorigenesis. Moreover, osteoblast-specific deletion of *Dlg2* in a clinically relevant genetically engineered mouse model leads to acceleration of OS development, establishing DLG2 as a critical determinant of OS. This widely applicable cross-species approach serves as a platform to expedite the search of cancer drivers in rare human malignancies, offering new targets for cancer therapy.

## Introduction

Extensive genomic alterations in orphan cancers make it difficult to identify driver mutations. Osteosarcoma (OS) presents a major challenge due to its chaotic genome and limited patient populations. Cytogenetic and genomic studies report a high degree of chromothripsis, chromosomal instability, copy number aberrations, and structural variations in OS [1–5]. Multiple patient cohorts have undergone aCGH amounting to ~100 OS genomes, and whole-genome sequencing was applied to 34 OS cases [6]. OS, the most common bone malignancy in adolescents, severely compromises the quality of life in children and adolescents. Further, despite improvements in orthopedic surgery and combination chemotherapy over the past 4 decades that have enhanced patient outcome, the 5-year survival rate of OS has plateaued at ~65% [7]. New strategies are urgently required to uncover the molecular determinants of this cancer in order to design novel targeted therapies.

Naturally occurring canine cancers offer a cross-species approach to understanding the pathogenesis of human cancers due to the considerable homology between dog and human genomes. OS is one of the most frequent malignancies in dogs and shares remarkable clinical similarity with the human disease including radiological and histological features, and the pattern of metastatic spread. Compared to mice, dogs are relatively out-bred and share the same living environment as humans. Publication of high-resolution dog reference genomes now permit a detailed genomic comparison between these species [8–11]. We created a novel comparative oncogenomics platform in this study, compiling high-resolution human and newly generated canine OS aCGH data, to identify known and novel drivers in bone cancer. Discs large homolog 2 (DLG2) was highly mutated in both human and dog, and was validated as a tumor suppressor through a workflow that spanned human and canine cell lines, and a clinically relevant murine model of OS.

## Results and discussion

**Fig. 1:**
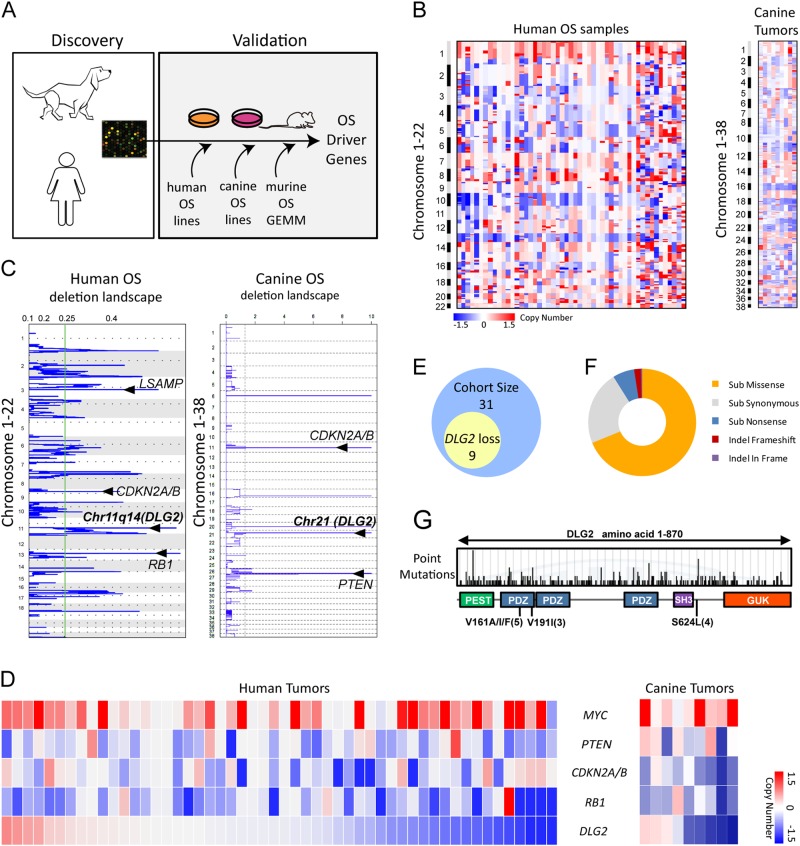
Whole-genome landscape of CNAs in human and canine OS identifies DLG2 locus. **a** A schematic of cross-species oncogenomics strategy begins with comparison of curated human and new dog aCGH data on OS. Recurrent events serve as a filter to limit the number of candidate genes and selected new gene candidate undergoes biological validation through gain-of-function and loss-of-function studies designed to assess its impact on tumorigenic properties in vitro and oncogenic or tumor suppressor function in vivo. **b** Segmented CNA profiles of 52 human OS genome (left panel) and 9 canine OS genome (right panel) were mapped. *X*-axis presents individual samples. *Y*-axis lists chromosomal locations from Chr1 to Chr22 for human, or Chr1 to Chr38 for dog. **c** Significant deletion regions calculated by GISTIC across the human and canine OS genome. Important genes in the peaks are labeled. *X*-axis: significant score, *Y*-axis: chromosomal locations. **d** GRID heat map showing copy number status of top mutated genes. Samples are ordered by DLG2 status. **e** Of 31 human OS samples, 9 exhibited DLG2 deletion in an independent cohort. **f** Pattern of point mutations in DLG2 genes based on data from COMSIC. Most mutations result in amino acid changes. **g** Mutation hot spots across DLG2 amino acid sequences

To create a comparative oncogenomics platform (Fig. 1a), we selected a published dataset of 52 human OS samples which contain 40 tumor specimens and 12 OS cell lines (GSE12789). In parallel, 9 canine OS specimens representing a number of dog breeds were subjected to aCGH (Supplemental Table S1). Among the multiple tools used to segment the human and canine data, we found the greatest concordance between Circular Binary Segmentation (CBS) and Gain and Loss Analysis of DNA (GLAD); these were used to define the copy number profile [12, 13]. Segmented data were next analyzed by GISTIC (FDR *Q*-value < 0.1) to shortlist recurrent genomic regions for each species [14]. While candidate genes in human were directly produced by GISTIC, those in dog were pinpointed through cross species synteny matching of 38 canine chromosomes to the human reference genome, hg19. Human and canine OS genomes contained high levels of chromosomal instability marked by frequent whole chromosomal arm gains and losses (Fig. 1b). Mutational frequency of recurrent CNAs across samples showed that among the top eight highly significant genes, six were common between human and canine OS (Fig. 1c and Supplemental Fig. 1A), providing proof-of-principle for our approach. These included known oncogenes (*MYC*) and tumor suppressors (*CDKN2A/B*, *RB1, and PTEN*) indicating the high level of disease similarity and the overlapping mutational landscape across these species [15–18]. We recorded a novel focal deletion in Chr11q14 across multiple cases, harboring potential tumor suppressor gene(s) (Fig. 1c). Specifically, 42% (22/52; Chr11q14) of human OS and 55.6% (5/9; Chr21) of canine OS exhibited this focal loss (Supplemental Fig. 1B). This frequency is consistent with that reported for next-generation sequencing of 34 human OS cases (52.9%, 18/34) [6], although its biological significance was not pursued. We noted that *DLG2* is the only gene residing in the focal deletion in human (chr11: 83835663–84011662: 180 kb) and in dog (chr21:17457665–17858164: 400 kb). The relative distribution of *DLG2* deletion and other common alterations across human and dog OS is shown in Fig. 1d.

We then acquired an independent cohort of 31 human OS samples to examine the *DLG2* status using genomic qPCR (courtesy of Dr. David Malkin). Of 31 samples, 9 displayed *DLG2* deletion (29%; Fig. 1e), providing a validation in this independent patient cohort. Next, interrogation of the Catalogue of Somatic Mutations in Cancer (COSMIC) database exposed *DLG2* to be highly mutated in a variety of human cancers where most of the mutations resulted in drastic amino acid changes (Fig. 1f and Supplemental Table 2) [19]. DLG2 is a member of the Membrane-Associated Guanylate Kinase (MAGUK) protein family that is characterized by concatenations of PDZ, SH3 and GUK domains. These domains enable MAGUKs to function as scaffold proteins that orchestrate the assembly of multiple signal transduction networks [20]. The mammalian DLG family of proteins are primarily known for their role in epithelial polarity and polarity during cell division [21]. Several mutational hotspots were seen: V161 and V191 within the first PDZ domain; and S624 in the connector sequence adjacent to the SH3 domain (Fig. 1g).

**Fig. 2:**
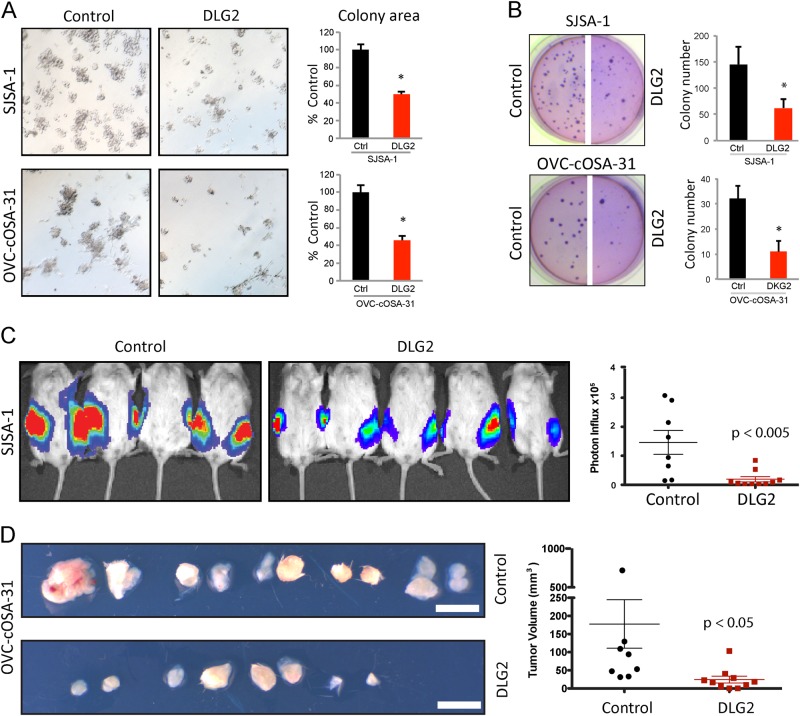
DLG2 restoration hinders cell and tumor growth in in vitro 3D cultures and xenograft experiments. **a** Representative pictures of colonies in 3D-matrigel. DLG2 re-expression induced a significant reduction in both colony number and size. Quantification of colony area is shown on the right. **b** Representative soft agar images and their respective quantifications. DLG2 restoration reduces colony forming capacities. **c** NOD-SCID mice injected with luciferase- expressing SJSA-1 parental (left) and DLG2 (right) cells imaged at 3 weeks post injection (*n* = 10 per group). Photon influx value was used as a surrogate marker for tumor size. Photon influx of each tumor was quantified and average values are shown on the right. **d** Tumors taken from NOD-SCID mice 4 weeks after innoculation with OVC-COSA-31 parental (left) and DLG2 (right) canine OS cells (*n* = 10 per group). Tumor size was calculated and is shown on the right. White bar represents 1 cm. Values shown are the mean ± SD of three separate determinations. *P < 0.05 by two-tailed Student’s *t* test

To examine the impact of restoring DLG2 expression on tumorigenic properties, we sought relevant human and canine cell lines that naturally harbored DLG2 deletions. Data from GSE12789 and GSE36003 revealed that *DLG2* was deleted in SAOS-2, SJSA-1 and 2 primary OS cell lines (HuO-3N1 & HuO9), while *DLG2* loss was also detected by aCGH analysis in a canine OS cell line that we generated in house (OVC-cOSA-31); Supplemental Fig. 1C). Consistently, endogenous DLG2 protein expression was very low in these cells (Supplemental Fig. 1D). A human DLG2 expression vector was then constructed for the dominant, longest isoform1 (Supplemental Fig. 1D). DLG2 expression did not affect proliferation in normal or low-serum conditions (Supplemental Fig. 2A). However, when tested in anchorage-independent soft agar and 3D-Matrigel assays, DLG2 overexpression in SJSA-1 or OVC-COSA-31 cells significantly reduced their colony formation capacity (Fig. 2a, b). HuO9 and SAOS-2 did not grow in anchorage-independent conditions. We then tested the effect of DLG2 restoration in vivo. Luciferase-tagged [22], parental SJSA-1 and OVC-COSA-31 cell lines formed xenografts when injected subcutaneously into NOD-SCID mice, whereas DLG2 restored cell lines showed slower xenograft growth, resulting in smaller tumors (Fig. 2c, d).

**Fig. 3:**
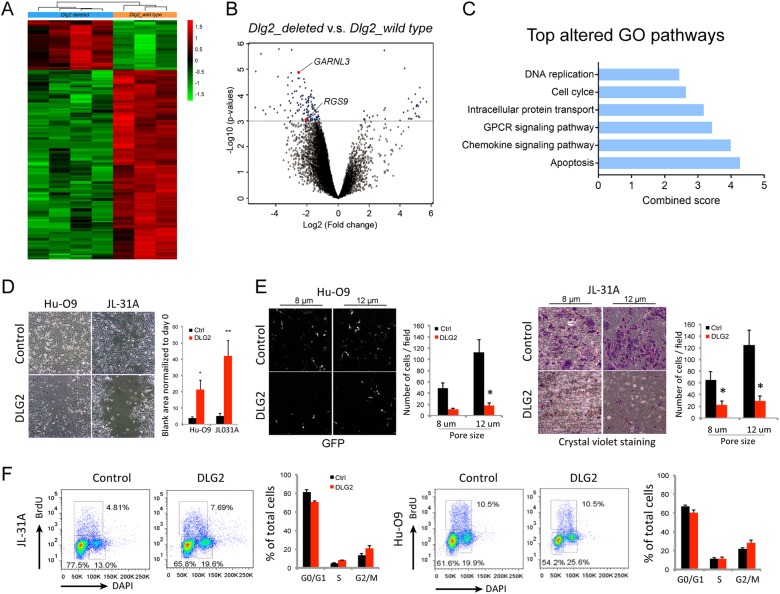
DLG2 is involved in small GTPase activity and its restoration reduces migratory capacities. **a** Differentially expressed probes/genes in Dlg2-deleted (*n* = 4, blue) vs. Dlg2-wild-type (*n* = 3, orange) groups. **b** Volcano plots show differential probes/genes identified using *T*-test with FDR correction. **c** Top altered pathways were analyzed using Enrichr online tool. G- protein-coupled receptor protein signaling pathway was among the top significant pathways. **d** Representative images of HuO9 and OVC-COSA-31 control and DLG2-re-expressing cells 24 h after a scratch-wound. Quantification of blank areas is shown on the right. **e** Representative images of Hu-O9 and OVC-COSA-31 cells 24 h after seeding in transwells. Transwell insert pore size of 8 and 12 μm were used. Quantification of cells that migrated through the transwell is shown on the right. **f** Cell cycle analysis showing DLG2 induced cell cycle changes in OVC-COSA-31 and Hu-O9 cells cultured in ultra-low attachment plate. DAPI (*x*-axis) was used to determine DNA content and BrdU (*Y*-axis) was used to determine nucleotide uptake. DLG2 restoration resulted in significant accumulation of cells in G2/M and S phases. Quantification of cells at specific stages of the cell cycle is shown on the right. Values shown are the mean ± SD of three separate determinations. *P < 0.05 by two-tailed Student’s *t* test

We next investigated the gene expression profiles associated with DLG2 deletion in canine OS. Microarrays generated from 7 aCGH-matched canine primary tumors (4 *DLG2* deleted, 3 *DLG2* wild type) showed 167 differentially expressed probes/genes (DEGs) in the two groups (Fig. 3a, b, Supplemental Table 3). Interestingly, G protein-coupled receptor (GPCR) signaling was one of the top altered pathways (Fig. 3c). The functional interactions between PDZ domain-containing proteins such as DLG1 and DLG4, and GPCRs have been implicated in many diseases including cancer [23]. Indeed, biological process prediction of DLG2 using Integrative Multi-species Prediction (IMP) database (http://imp.princeton.edu) showed that DLG2 is also involved in GPCR signaling pathway (GO term ID: GO:0086103, GO:0007186, GO:0008277). Importantly, GTPase-activating proteins RGS9 (Regulator of G-protein signaling 9) [24] and GARNL3 (GTPase activating Rap/RanGAP domain-like 3) [25] called as GAPs were among the most downregulated genes between *DLG2*-deleted and *DLG2*-wild-type groups (Fig. 3b). GAPs are a family of regulatory proteins that bind to activated G proteins and result in “switching off” of GTPase signaling [26]. Therefore, RGS9 and GARNL3 downregulation in DLG2-deleted tumor may lead to increased GTPase signaling activity.

Small GTPase signaling pathway, such as Rho and Ran, has been previously implicated in cytoskeletal re-organization and migration [27]. We therefore performed scratch-wound and transwell migration assays. DLG2-expressing cell lines showed significantly decreased motility in both assays compared to their respective parental control cells (Fig. 3d, e). This phenotype is in agreement with our gene expression data indicating a negative regulatory role for DLG2 in the GTPase signaling pathway, which in turn influences human and canine OS cell migration. Since GTPase signaling also orchestrates multiple stages of the cell cycle, we asked whether DLG2 status affects the cell cycle using flow cytometry [28]. We observed more cells in either G2/M or S phases when DLG2-re-expressing cells were grown under low-attachment conditions (Fig. 3f) but not when grown as a monolayer (data not shown). This observation together with slower proliferation under 3D, but not 2D conditions (Fig. 2a, b, and Supplemental Fig. 2A) suggests that DLG2 participates in regulating cell cycle progression. It is likely that the tumor suppressor function of DLG2 is mediated in part through modulating the small GTPase signaling pathway.

**Fig. 4:**
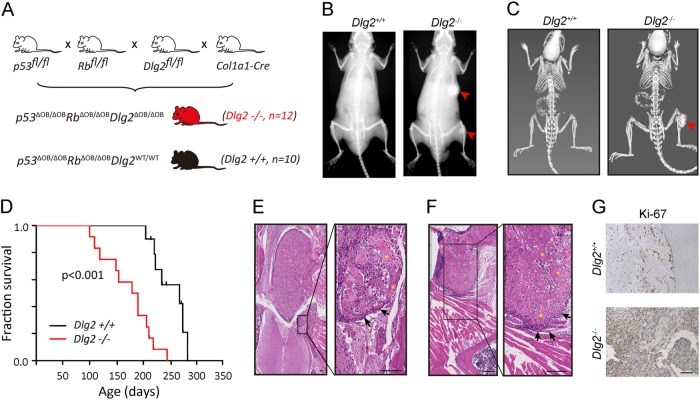
Osteoblast-specific Dlg2 knockout reduces overall survival and accelerates tumor onset. **a** Breeding strategy for generating osteoblast-specific compound deletion of Dlg2, p53, and Rb1. **b** Representative X-ray and **c** MicroCT images. At 22 weeks of age, Dlg2 wild type mice show no visible signs of tumor while Dlg2 conditional knockout mice developed tumors on tibia and rib. **d** Kaplan–Meier curve of mice with different Dlg2 status. Dlg2 homozygous conditional knockout shortens overall survival compared to Dlg2 wild type. **e** H&E images of a thoracic vertebra osteosarcoma. At higher magnification, osteosarcoma cells (arrows) and osteoid deposition (asterisk) were seen invasive fronts. **f** H&E images of a femoral osteosarcoma located on the Ilium. Note the plump tumorous osteoblasts (arrows) and massive osteoid deposition (asterisk). **g** Ki-67 staining of indicated tumor tissues. Scale bar: 100 μm

The single Drosophila DLG homolog is a tumor suppressor [29]. To examine whether mammalian DLG2 functions as a tumor suppressor, we utilized a genetically engineered mouse model (GEMM) of OS. *RB* and *TP53* mutations are the most common events in OS and germline mutations also predispose humans to this cancer. It has been shown that combined osteoblast-specific deletion of *p53* and *Rb*, achieved through the Collagen Type 1 Alpha-1 (*Col1a1*) promoter-driven *Cre* recombination in mice leads to spontaneous OS development [30, 31]. We bred *Dlg2*-floxed mice into the above clinically relevant model to generate triple osteoblast-specific deletions (*p53*^ΔOB/ΔOB^*Rb*^ΔOB/ΔOB^*Dlg2*^ΔOB/ΔOB^: *Dlg2*^−/−^, Fig. 4a)*. Dlg2* homozygous deletion significantly accelerated tumor development and shortened survival in mice (Fig. 4b–d). Specifically, the median lifespan for the *Dlg2*^−/−^ cohort was 184 days vs. 239 days in the control group (*p53*^ΔOB/ΔOB^*Rb*^ΔOB/ΔOB^*Dlg2*^WT/WT^: *Dlg2*^+/+^). The anatomical distribution and histological features of OS tumors developed in *Dlg2*^−/−^ mice remained comparable to littermate controls. The abundant osteoid deposition at the invasive the front of thoracic vertebra osteosarcoma (Fig. 4e) illustrates the aggressive nature, osteoblastic nature of tumors developed in these mice. Histological features of osteosarcoma, such as plump tumorous osteoblasts and copious amount of osteoid were also seen in tumors that developed at different sites in the *Dlg2-*null mice (Fig. 4f). Moreover, tumor cells derived from *Dlg2*-deficient mice were more proliferative than those of wild-type mice, as indicated by Ki-67 staining (Fig. 4g).

Altogether, through cross-species oncogenomics we have uncovered *DLG2* deletion to be a highly frequent event in human (42%) and dog (56%) OS. We extended our cross-species approach to molecular and functional analyses of DLG2 by utilizing pertinent human and dog OS cell lines as well as p53/Rb-driven OS GEMM. These series of studies establish DLG2 as a bone tumor suppressor and provide insight into its involvement in GTPase signaling and regulation of cell cycle and proliferation in a 3D tissue culture model. This work presents a blue print for cross-species comparative genomics platform as a useful tool for driver gene discovery in rare human cancers.

## Methods

### aCGH analysis

Published human OS genomic datasets on Gene Expression Omnibus (GEO) were selected (GSE12789). Canine OS specimens were obtained from University of Guelph veterinarian clinics. Genomic DNA was extracted using phenol chloroform and treated with RNaseA (Invitrogen) for 1 h at 37 °C. Canine aCGH assay was performed by SurePrint G3 Canine CGH Microarray 4 × 180 K (Agilent) according to manufacturers’ specifications at the University Health Network Microarray Centre. CBS and GLAD were used for segmentation of the genome and GISTIC was used to determine the common altered region and the significance of genetic events. Canine aCGH data were deposited in the GEO database (GSE111637).

### Gene expression microarray analysis

Total RNA from canine OS tumors was prepared using the RNeasy mini-kit (QIAGEN), RNA concentration and integrity were assessed using Nanodrop 2000 and Bioanalyzer (Thermo Scientific and Agilent). Gene expression microarray on Canine (V2) Gene Expression Microarray, 4 × 44k (Agilent) were performed at the University Health Network Microarray Centre. Multi-array average (RMA) normalization was used across the sample sets and batch correction was performed. Differentially expressed genes were identified using *T*-test with FDR correction (*P* < 0.05 and FDR < 0.1) or Samtools. Analyses were performed using R (version 2.82), Agilent Genespring GX (version 11) or BRBArrayTools. Gene ontology and pathway analysis were performed using Enrichr. Total Ensemble genes were used as background. Microarray data were deposited in the GEO database (GSE111638).

### Mice

Osteoblast-specific Dlg2 knockout OS mouse model (*p53*^*ΔOB/ΔOB*^ *Rb*^*ΔOB/ΔOB*^*Dlg2*^*ΔOB/ΔOB*^) was generated by breeding *Dlg*^*flox/+*^ (*Dlg2*^*tm1a(EUCOMM)Wtsi*^, obtained from Wellcome Trust Sanger Institute) with *Col1a1-Cre-p53*^*flox/flox*^*Rb*^*flox/flox*^ (*n* = 12). *p53*^*ΔOB/ΔOB*^*Rb*^*ΔOB/ΔOB*^*Dlg2*^*wt/wt*^ littermates (*n* = 10) were used as the control. X-ray and MicroCT scan were carried out as previously reported [28]. For xenograft experiments, 8-week-old female recipient non-obese diabetic/severe combined immunodeficiency (NOD-SCID) mice were obtained from Charles River Laboratories. Mice were assigned to experimental groups by stratified randomization according to body weight. OVC-COSA-31 and luciferase-expressing SJSA-1 cells were injected subcutaneously on both flanks (*n* = 10 per group) and monitored weekly for 4–8 weeks by electronic caliper or bioluminescence imaging using Xenogen IVIS Imaging System 100 (STTARR facility, UHN). Investigators who measured and analyzed the samples were blind to the group information.

### Statistics

Two-tailed Student’s *t*-test was used to determine if two sets of normally distributed data are significantly different from each other. Biological replicates were performed independently at least three times. Log-rank test was performed for survival curves. For all studies, *P* < 0.05 was considered significant. Specialized analyses for bioinformatic data, including aCGH analysis and gene expression microarray analysis, were performed using dedicated tools mentioned above.

### Study approval

All animal protocols were approved by the University Health Network Animal Care Committee and performed in accordance with the standards of the Canadian Council on Animal Care. Mice were housed in the Princess Margaret Hospital Animal Care Facility. User protocol AUP 849.1 was strictly followed. Written informed consent was obtained from the patients or their guardians before sample collection.

## Electronic supplementary material

Supplemental data
