# Supplementary material for: Cross-species genomics identifies DLG2 as a tumor suppressor in osteosarcoma
Source: Oncogene. 2018 Aug 9;38(2):291–8. doi: 10.1038/s41388-018-0444-4 (PMC6756098; doi:10.1038/s41388-018-0444-4)
Supplement: Supplementary file 1 — Supplemental data [file 41388_2018_444_MOESM1_ESM.pdf]

Supplemental data

Methods

Cell culture: U2OS, SAOS-2 and SJSA-1 cells were obtained from ATCC, while HuO-3N1 and HuO9 were from the RIKEN Institute. Canine OS cell line OVC-cOSA-31 was established in University of Guelph. Cells were cultured according to the provider's specific protocol. Viable cell quantifications were done using Celltiter-Glo or hemocytometer.

Real-time PCR: Genomic DNA was isolated from human osteosarcoma tissues using DNeasy Mini Kit (Qiagen). Real-time PCR was carried out using Perfecta SYBR Green Super mix (Quantabio) according to the manufacturer's protocol using Applied Biosystems 7500 PCR system. Human genomic DNA (Promega) was used as the control sample. The PCR primers used here were as follows: DLG2-F: 5'-TGTTGAGGATGTGGTGGAGA-3', DLG2-R: 5'-TTGTACTGGCCGGCTTCTAT-3'; RRP30-F: 5'-GCAAGTAAGTTTCTCCGAATCC-3', RPP30-R: 5'-GCGCAGAGCCTTCAGGT-3' (internal control). Relative copy number was compared to control, and calculated as $2^{-\Delta\Delta CT}$. Value less than 0.5 was determined as deletion.

Lentiviral transduction: DLG2 open reading frame expression (EX-Z6752-Lv201) and empty control clones were obtained from Genecopoeia. Lentivirus was produced using Lenti-Pac HIV Expression Packaging kit (Genecopoeia). To generate stable cell lines, osteosarcoma cells were transduced with the indicated lentiviruses (MOI = 10~20) and selected with 1 µg/ml puromycin.

Western blot: The analysis was performed by standard procedures. Antibodies against DLG2 (1:1000, AB5168-200UL, Millipore) and β-actin (1:1000, #3700, Cell Signaling Technology) were used. The protein bands were detected by the enhanced chemiluminescence detection system.

3D Matrigel colony formation assay： 200μl of Matrigel (BD Biosciences) was added to each well of a 24-well tissue culture plate and allowed to solidify for 1 hour at 37°C. 2X10^4 cells were suspended in 200ul 20% Matrigel in culture medium and seeded onto Matrigel-coated wells in triplicate. Plates were then incubated at 37 °C for 30 min to allow the Matrigel to solidify, and culture medium then was added. Cells were allowed to proliferate for 2–3 weeks and to form colonies. These were imaged and quantified under a phase-contract microscope.

Scratch-wound and transwell migration assay: Scratch-wound assays were carried out according to standard protocols. Cells were cultured for 24 hours to monitor migration. The gap distance was quantitatively evaluated using Image J software. Transwell migration assays were carried out on 24-well transwell insert（BD Biosciences), 10% FBS was used as an attractant and cells were then cultured for 24 hours.

Histology and immunohistochemistry: Organs or tumor samples were dissected, washed thoroughly with PBS, fixed with 4% paraformaldehyde (PFA) overnight at 4°C, decalcified in 20% EDTA (pH 7.4), and then embedded in paraffin. Sections (5 μm) were prepared and stained with hematoxylin & eosin (H&E) or Masson-Trichrome by Toronto Centre of Phenogenomics (TCP) pathology laboratory via routine protocols.

Cell cycle analysis by flow cytometry: BD Pharmingen BrdU Flow Kits and in-house flow cytometry protocol were followed. Briefly, BrdU was added to cells at a final concentration of 10 μM for 1-3 hours depending on the cell line. Flow cytometry preparation was performed using standard protocols. Data collection was done on BD Fortessa II and analysis was done using FlowJo Version 7.65 or Version VX.

A

Most frequently mutated genes

Human	Canine
RB1	CDKN2A/B
TP53	MYC
MYC	TP53
CDKN2A/B	PTEN
PTEN	**DLG2**
LSAMP	KIT
RUNX2	MDM2
**DLG2**	RUNX2

B

DLG2 Deletion in OS

	Human	Canine
Total	52	9
Deleted	22	5
%	42.3	55.6

C

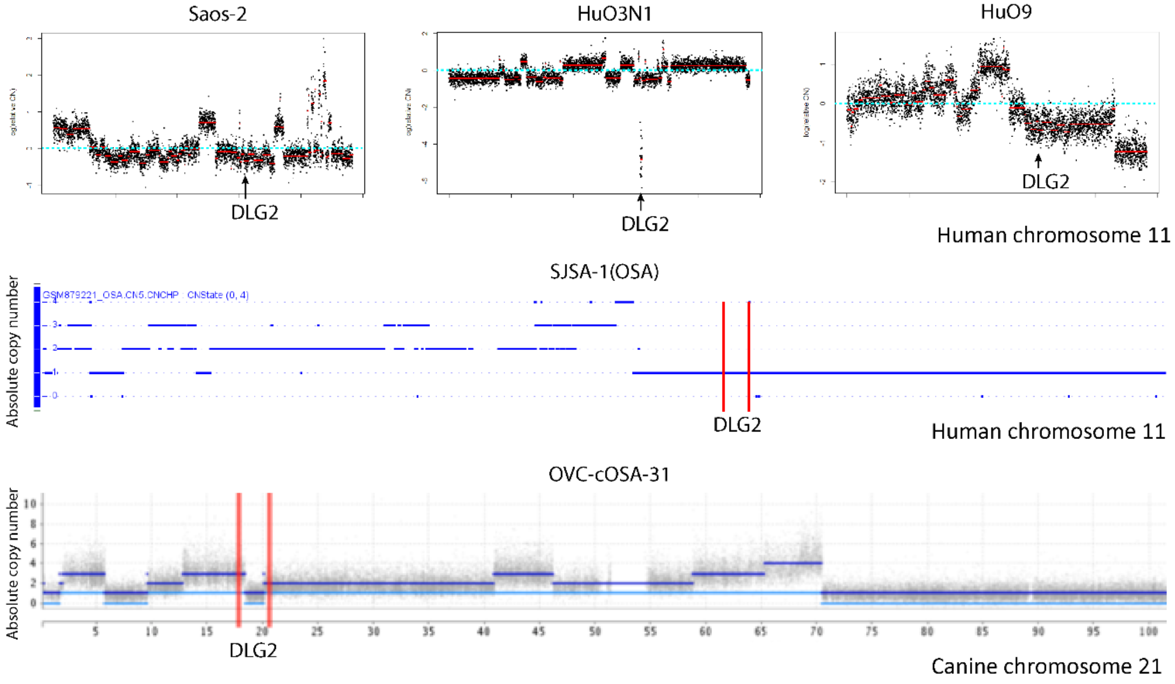

D

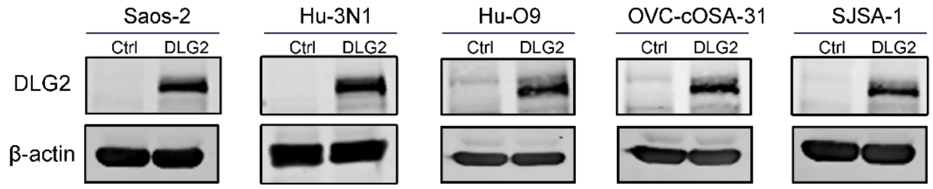

Supplemental Figure 1. (A) 6 of the 8 most frequently altered genes in osteosarcoma are common to both human and dog. Note DLG2 is among the top genes in both species. (B) Deletion frequencies of DLG2 in human and canine OS tumors. (C) Copy number profile of human and canine OS cell lines that are selected based on their DLG2 genomic status. Cell lines used in experiments exhibit DLG2 deletion. (D)Western blot analysis of selected cell lines with endogenous (Ctrl) or DLG2 overexpression.

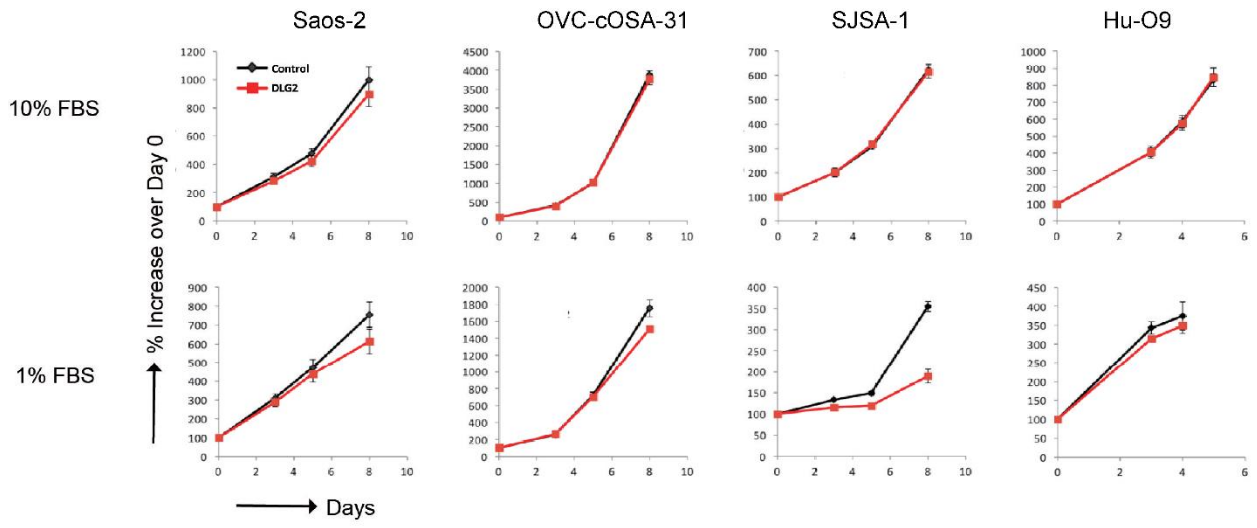

Supplemental Figure 2. (A)DLG2 restoration does not induce proliferation changes under 2D culture conditions. Top panel shows cell lines cultured with 10% FBS, bottom panel with 1% FBS.

Supplemental Table 1

Clinical features of the breeds of dogs employed in this study

Sample Number	Breed	Age	Sex	Survival Time
1	Rottweiler	7 yrs	MC	~6-7 months
2	Mixed Breed (German Shepherd/Collie/X)	8 yrs	MC	~12 months
3	Boxer	12 yrs	FS	~2-4 months
4	Mixed Breed (Labrador Retriever/X)	10 yrs	FS	~4-5 months
5	Rottweiler X (Canine Mixed Breed)	9 yrs	FS	~7 months
6	Greyhound	6 yrs	MC	~11 months
7	Bull Mastiff	8 yrs	MC	~6 months
8	Great Pyrenees	7 yrs	FS	~13 months
9	Great Pyrenees	5 yrs	MC	~5 months

Supplemental Table 2

DLG2 point mutations in various cancer genomes. Most mutations resulted in amino acid property changes.

a. *DLG2* mutations frequency in cancer

Tumor Type	# of Samples Analyzed	# of Mutation observed
Ovary	3	3
Pancreas	24	2
Breast	11	0
Pancreas	22	0
Skin	2	2
Biliary tract	2	0
Upper AG tract	3	3
Colon	11	0
CNS	22	0
Total	101	11

b. *DLG2* nonsynonymous mutations in cancer

mRNA	Protein	Before	After
c.2075G>A	p.R692Q	Positive	Polar
c.2158G>A	p.E720K	Negative	Positive
c.962C>T	p.P321L	Special	Hybrophobic
c.2287A>G	p.R763G	Positive	Polar
c.481G>T	p.V161F	Hybrophobic	Aromatic
c.491G>T	p.S164I	Polar	Hydrophobic
c.1589_1590CC>TT	p.S530F	Polar	Aromatic

Supplemental Table 3

167 differentially expressed probes/genes between DLG2 deleted and wild type canine primary tumors

No.	Probe ID	Fold change	Symbol	Name	EntrezID	Accession	p-value
1	A_11_P079441	0.025	NPY	neuropeptide Y	475257	XM_532492	1.18E-05
2	A_11_P0000016871	0.025					0.000299
3	A_11_P164793	0.032					2.50E-06
4	A_11_P150478	0.035					0.000387
5	A_11_P0000016604	0.039					0.000551
6	A_11_P0000035706	0.048					0.00028
7	A_11_P161333	0.058					0.000341
8	A_11_P182068	0.066					0.000472
9	A_11_P126441	0.068	FLRT3	fibronectin leucine rich transmembrane protein 3	477149	XM_534339	1.92E-05
10	A_11_P120626	0.071					1.60E-06
11	A_11_P079656	0.076	LRRN3	leucine rich repeat neuronal 3	482406	XM_539523	0.000243
12	A_11_P0000031395	0.093					0.000394
13	A_11_P091981	0.1					4.06E-05
14	A_11_P0000023452	0.11	SCG5	secretogranin V (7B2 protein)	478249	XM_535423	0.000737
15	A_11_P0000022534	0.11					1.99E-05
16	A_11_P0000042014	0.11					9.20E-05
17	A_11_P000009568	0.11					0.000168
18	A_11_P0000045	0.12	ENPP2	ectonucleotide pyrophosphatase/phosphodiesterase 2	482026	XM_539147	0.000396
19	A_11_P207533	0.12					1.36E-05
20	A_11_P156838	0.12					0.0002
21	A_11_P134231	0.13	BVES	blood vessel epicardial substance	481944	XM_539065	8.51E-05
22	A_11_P0000039966	0.13					1.70E-06
23	A_11_P189798	0.13					2.85E-05
24	A_11_P000003463	0.13					0.000126
25	A_11_P0000040386	0.13					0.000636
26	A_11_P102221	0.14	PCDHB14	protocadherin beta 14	487175	XM_844094	7.11E-05
27	A_11_P067266	0.15					0.00049
28	A_11_P0000026871	0.16	GPR85	G protein-coupled receptor 85	482410	XM_539527	0.000196
29	A_11_P183668	0.16					6.93E-05
30	A_11_P192748	0.16					0.000383
31	A_11_P119191	0.17	GARNL3	GTPase activating Rap/RanGAP domain-like 3	480718	XM_857841	1.34E-05
32	A_11_P112196	0.17	TTYH1	tweety homolog 1 (Drosophila)	476386	XM_533589	1.25E-05
33	A_11_P0000016845	0.17					2.21E-05
34	A_11_P0000011239	0.17					0.000243
35	A_11_P180248	0.17					0.000411
36	A_11_P083571	0.18					2.20E-05
37	A_11_P137766	0.18					4.57E-05

38	A_11_P066316	0.18					0.000365
39	A_11_P0000034033	0.19	IGF2BP1	insulin-like growth factor 2 mRNA binding protein 1	491064	XM_003435261	0.000287
40	A_11_P210608	0.19	TNNI3	troponin I type 3 (cardiac)	403566	NM_001003041	0.00022
41	A_11_P0000021443	0.19					0.000114
42	A_11_P097266	0.2	RBFOX1	RNA binding protein, fox-1 homolog (C. elegans) 1	609116	XM_003434859	0.000837
43	A_11_P083566	0.2					0.000102
44	A_11_P0000023967	0.2					0.00014
45	A_11_P051471	0.2					0.000885
46	A_11_P0000029082	0.21					0.000104
47	A_11_P106986	0.21					0.000246
48	A_11_P051506	0.21					0.000528
49	A_11_P051466	0.21					0.00092
50	A_11_P126166	0.22	LOC484934	zinc finger protein 709-like	484934	XM_542050	2.36E-05
51	A_11_P183013	0.22					3.15E-05
52	A_11_P123506	0.22					0.000372
53	A_11_P187748	0.22					0.000513
54	A_11_P136846	0.23	TRIM37	tripartite motif containing 37	480575	XM_537697	0.00099
55	A_11_P100896	0.23					5.22E-05
56	A_11_P0000017143	0.23					0.000995
57	A_11_P064171	0.24	TOX	thymocyte selection-associated high mobility group box	486964	XM_544093	0.000915
58	A_11_P0000019766	0.24					0.000196
59	A_11_P0000013952	0.24					0.000146
60	A_11_P193928	0.24					0.000347
61	A_11_P177133	0.24					0.000453
62	A_11_P000002641	0.24					0.000833
63	A_11_P0000032744	0.24					0.000867
64	A_11_P081231	0.25	RGS9	regulator of G-protein signaling 9	490902	XM_548024	0.00092
65	A_11_P196063	0.25					0.000461
66	A_11_P0000017027	0.25					0.00018
67	A_11_P109051	0.25					0.00038
68	A_11_P0000024127	0.25					0.000768
69	A_11_P0000021356	0.25					0.000812
70	A_11_P173248	0.26	FKBP4	FK506 binding protein 4, 59kDa	477726	XM_534923	0.000194
71	A_11_P0000026525	0.26	HS3ST5	heparan sulfate (glucosamine) 3-O-sulfotransferase 5	481968	XM_539089	0.000973
72	A_11_P0000041559	0.26					0.000118
73	A_11_P153568	0.26					0.000138
74	A_11_P0000039024	0.26					0.000165
75	A_11_P129106	0.26					0.000274
76	A_11_P0000039793	0.26					0.000469
77	A_11_P127746	0.26					0.000593
78	A_11_P0000036794	0.26					0.000881
79	A_11_P0000018034	0.26					0.000953
80	A_11_P063851	0.27	LHPP	phospholysine phosphohistidine	486930	XM_544060	0.000158

				inorganic pyrophosphate phosphatase			
81	A_11_P0000029296	0.27					0.000254
82	A_11_P194038	0.27					0.00042
83	A_11_P178678	0.27					0.000579
84	A_11_P165623	0.27					0.000718
85	A_11_P0000022486	0.28	RBP1	retinol binding protein 1, cellular	477090	XM_534285	0.000117
86	A_11_P055691	0.29	LMX1A	LIM homeobox transcription factor 1, alpha	609061	XM_846259	0.000799
87	A_11_P0000013025	0.29					1.00E-04
88	A_11_P0000017802	0.29					0.000647
89	A_11_P109606	0.29					0.000929
90	A_11_P0000023052	0.3	HTR7	5-hydroxytryptamine receptor 7	477762	XM_534958	0.000571
91	A_11_P107231	0.3	NFIA	nuclear factor I/A	479552	XM_536691	6.69E-05
92	A_11_P109173	0.3	NTRK3	neurotrophic tyrosine kinase, receptor, type 3	609087	XM_846291	0.000979
93	A_11_P154218	0.3					0.000179
94	A_11_P0000015020	0.3					0.000564
95	A_11_P0000024245	0.31	RTKN2	rhotekin 2	479218	XM_536360	0.000226
96	A_11_P172868	0.31					0.000117
97	A_11_P000008616	0.31					0.000265
98	A_11_P0000041919	0.31					0.000266
99	A_11_P0000041691	0.31					0.000317
100	A_11_P207618	0.31					0.000793
101	A_11_P113571	0.31					0.000887
102	A_11_P0000027232	0.32	DDX43	DEAD (Asp-Glu-Ala-Asp) box polypeptide 43	611029	XM_848647	0.000343
103	A_11_P116626	0.32	PCNA	proliferating cell nuclear antigen	477166	XM_534355	0.00088
104	A_11_P181218	0.32					0.000599
105	A_11_P098321	0.32					0.000922
106	A_11_P0000024788	0.33	ATF7IP2	activating transcription factor 7 interacting protein 2	479852	XM_536977	0.000252
107	A_11_P0000019679	0.33					0.000128
108	A_11_P000006209	0.34					0.000126
109	A_11_P0000011243	0.34					0.000316
110	A_11_P055711	0.35	HSD17B7	hydroxysteroid (17-beta) dehydrogenase 7	609364	XM_846605	0.000847
111	A_11_P000001652	0.35					0.000238
112	A_11_P0000014425	0.35					0.000374
113	A_11_P187463	0.35					0.000375
114	A_11_P000006343	0.35					0.000552
115	A_11_P000003665	0.35					0.000875
116	A_11_P0000030689	0.36	HNF4G	hepatocyte nuclear factor 4, gamma	487005	XM_003433668	0.000925
117	A_11_P089591	0.36	MCM5	minichromosome maintenance complex component 5	610519	XM_848041	0.000883
118	A_11_P207463	0.37					0.000408
119	A_11_P000004813	0.37					0.000857
120	A_11_P128496	0.38	TOM1L1	target of myb1 (chicken)-like 1	491093	XM_847759	0.000761
121	A_11_P000005752	0.38					0.000448

122	A_11_P217428	0.38					0.000689
123	A_11_P0000017227	0.38					0.000704
124	A_11_P000003008	0.38					0.000828
125	A_11_P217163	0.39					0.000867
126	A_11_P0000039429	0.4					0.000703
127	A_11_P159463	0.4					0.000725
128	A_11_P0000011879	0.4					0.00088
129	A_11_P0000028408	0.41	EPS8L1	EPS8-like 1	484302	XM_541418	0.000601
130	A_11_P0000038562	0.41					0.000441
131	A_11_P174643	0.42					0.000675
132	A_11_P176368	0.44					0.000838
133	A_11_P058421	3.13					0.000877
134	A_11_P0000019663	3.17	CD8A	CD8a molecule	403157	NM_001002935	0.000419
135	A_11_P050776	3.25					0.000839
136	A_11_P0000020101	3.28	CCL4	chemokine (C-C motif) ligand 4	448786	NM_001005250	0.000931
137	A_11_P058996	3.65	CXCL13	chemokine (C-X-C motif) ligand 13	608156	XM_845089	0.000616
138	A_11_P114341	4.05	DMD	dystrophin	606758	NM_001003343	0.000736
139	A_11_P094216	4.74					0.000654
140	A_11_P0000024409	5.72					0.000599
141	A_11_P094241	7.73					0.000373
142	A_11_P0000030842	7.84	GZMA	granzyme A	487207	XM_544335	1.80E-06
143	A_11_P094231	8.11	LOC607543	Ig heavy chain V-III region VH26-like	607543		0.000316
144	A_11_P0000025562	8.14					0.000828
145	A_11_P196708	15.79					0.000952
146	A_11_P054741	18.6					0.000472
147	A_11_P060756	19.45					9.10E-06
148	A_11_P101211	22.41					0.000395
149	A_11_P054732	23.07					0.000826
150	A_11_P145753	23.2					0.000136
151	A_11_P000001229	24.7					0.000823
152	A_11_P054921	26.65					0.000184
153	A_11_P060584	26.69	LOC612135	uncharacterized LOC612135	612135		0.000828
154	A_11_P060571	28.87	LOC612122	uncharacterized LOC612122	612122	XM_849848	0.000333
155	A_11_P053591	31.26					9.13E-05
156	A_11_P060626	31.4	LOC491494	Ig lambda chain V region 4A-like	491494		0.000518
157	A_11_P054646	32.85					0.000646
158	A_11_P060611	32.96					0.000272
159	A_11_P138911	33.61					0.00024
160	A_11_P0000022913	34.85					0.000251
161	A_11_P094171	35.59					0.000267
162	A_11_P060656	35.72	LOC486384	Ig kappa chain V region Mem5-like	486384		0.000655
163	A_11_P054726	37.62					0.000721
164	A_11_P060716	38.31	LOC607200	Ig lambda chain V-I region BL2-like	607200		5.10E-05
165	A_11_P054711	39.59					0.000186

166	A_11_P054596	44.45					0.000135
167	A_11_P054606	51.61					0.000181
